# *TelNet* - a database for human and yeast genes involved in telomere maintenance

**DOI:** 10.1186/s12863-018-0617-8

**Published:** 2018-05-18

**Authors:** Delia M. Braun, Inn Chung, Nick Kepper, Katharina I. Deeg, Karsten Rippe

**Affiliations:** Division of Chromatin Networks, German Cancer Research Center (DKFZ) & Bioquant, 69120 Heidelberg, Germany

**Keywords:** Telomere maintenance, Telomerase, Alternative lengthening of telomeres (ALT), Pathway analysis

## Abstract

**Background:**

The ends of linear chromosomes, the telomeres, comprise repetitive DNA sequences in complex with proteins that protects them from being processed by the DNA repair machinery. Cancer cells need to counteract the shortening of telomere repeats during replication for their unlimited proliferation by reactivating the reverse transcriptase telomerase or by using the alternative lengthening of telomeres (ALT) pathway. The different telomere maintenance (TM) mechanisms appear to involve hundreds of proteins but their telomere repeat length related activities are only partly understood. Currently, a database that integrates information on TM relevant genes is missing.

**Description:**

To provide a resource for studies that dissect TM features, we here introduce the *TelNet* database at http://www.cancertelsys.org/telnet/. It offers a comprehensive compilation of more than 2000 human and 1100 yeast genes linked to telomere maintenance. These genes were annotated in terms of TM mechanism, associated specific functions and orthologous genes, a TM significance score and information from peer-reviewed literature. This TM information can be retrieved via different search and view modes and evaluated for a set of genes as demonstrated for an exemplary application.

**Conclusion:**

*TelNet* supports the annotation of genes identified from bioinformatics analysis pipelines to reveal possible connections with TM networks. We anticipate that *TelNet* will be a helpful resource for researchers that study telomeres.

**Electronic supplementary material:**

The online version of this article (10.1186/s12863-018-0617-8) contains supplementary material, which is available to authorized users.

## Background

Telomeres, the ends of linear chromosomes, consist of repetitive DNA sequences bound by the shelterin protein complex [1, 2]. This protein assembly protects the DNA ends from degradation and accidental recognition as DNA double-strand breaks [3–5]. The progressive shortening of the telomere repeats that accompanies normal replication limits the number of cell divisions. Thus, it needs to be circumvented by cancer cells for unlimited proliferation. This is accomplished by activation of a telomere maintenance (TM) mechanism. It involves either the reactivation of the reverse transcriptase telomerase normally repressed in somatic cells via different mechanisms [6–9], or activation of the alternative lengthening of telomeres (ALT) pathway [10–13]. ALT activity in human cancer cells occurs via DNA repair and recombination pathways but details on the mechanism remain elusive. Thus, TM is a complex process that involves proteins that are part of the shelterin complex at telomere repeats [14, 15] or in close proximity [16, 17]. Factors that regulate transcription of telomere repeats and the activity of telomerase are also relevant [18, 19] as well as features of the ALT pathway like PML (promyelocytic leukemia) nuclear bodies at telomere repeats that are associated with a variety of proteins and referred to as APBs (ALT-associated PML nuclear bodies) [20–23]. Furthermore, studies of telomere shortening have linked a number of proteins to telomere crisis [24].

A well-studied model organism for telomere biology is the budding yeast *Saccharomyces cerevisiae* [25]. Several independent deletion screens with subsequent direct measurements of telomere length (TL) have identified a comprehensive list of yeast genes involved in TL regulation [26–28]. Since telomere structure and function are highly conserved between organisms, mammalian homologues exist for most of the genes identified in the various yeast screens. Thus, it is informative to relate TM phenotypes found in yeast to human homologues [29]. In *S. cerevisiae*, telomerase is constitutively active and its deletion leads to cellular senescence [30]. Survivor cells that overcome cellular senescence in the absence of telomerase use a mechanism based on homologous recombination for telomere elongation [31]. Interestingly, similar to ALT in human cells, so-called type II survivors are characterized by heterogeneous TLs [32, 33].

To compile telomere-relevant information several databases have been created: The *Telomerase database* (http://telomerase.asu.edu/overview.html) is a web-based tool for the study of structure, function, and evolution of the telomerase ribonucleoprotein [34]. It is a comprehensive compilation of information on the telomerase enzyme and its DNA substrate. In addition, *MiCroKiTS* (Midbody, Centrosome, Kinetochore, Telomere and Spindle; http://microkit.biocuckoo.org) provides information on the cellular localization of proteins relevant for cell cycle progression and also includes telomere proteins [35]. The *TeloPIN* (Telomeric Proteins Interaction Network) database was a collection of interaction data in human and mouse cells from available literature and GEO (gene expression omnibus) data [36] but it is no longer active. The same is true for the *TeCK database* that has been previously published as a collection of telomeric and centromeric sequences as well as telomerase, centromere and kinetochore binding proteins [37].

The above-mentioned databases cover telomere related information but lack an annotation of genes with respect to the TM mechanism. Accordingly, we here introduce the *TelNet* database as a compilation of information on TM relevant genes. *TelNet* currently comprises more than 2000 human, and over 1100 budding yeast genes that are involved in TM pathways. The annotation of these genes includes the classification of TM mechanisms (TMM) along with a significance score as well as TM specific functions and homology assignments between different organisms. Furthermore, links to the relevant literature sources are given. Thus, *TelNet* provides an integrative resource for dissecting TM networks and elucidating the alternative lengthening of telomeres pathway.

## Construction and content

### Implementation

The *TelNet* database was constructed using the Filemaker Pro software version 13. It is accessible at http://www.cancertelsys.org/telnet and is distributed with Filemaker server version 16 via its webdirect module. In addition, the *TelNet* webpage provides general information about *TelNet* as well as instructions on how to use it. Links to other databases and contact information are given as well.

### Data source

**Fig. 1:**
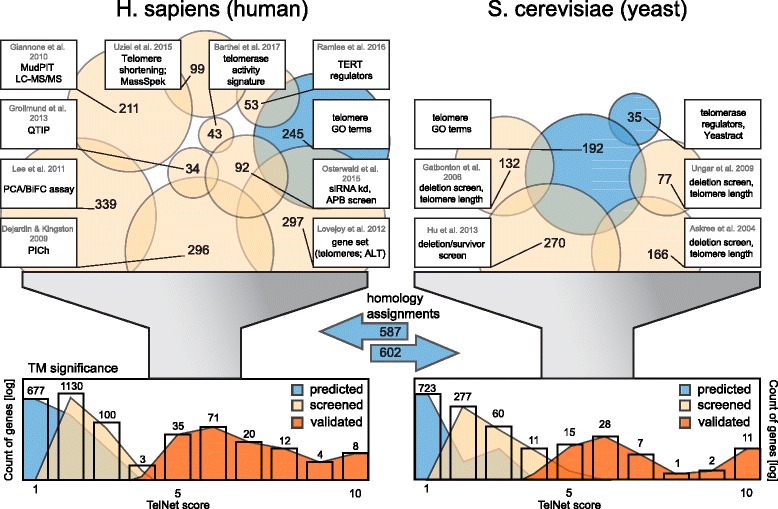
Data sources of TM genes included in *TelNet*. Selected screening studies and other references that served as sources for TM genes are shown. In total *TelNet* currently includes over 2000 human genes and more than 1100 budding yeast genes. Histograms of the *TelNet* scores are displayed for the complete gene sets per species and colored by their TM significance annotation. Color scheme: blue, predicted TM genes; beige, genes from screening studies; orange, validated genes

To compile an initial set of TM relevant genes, we selected screening studies on genes or proteins that play a role in telomere biology (Fig. 1, Table 1) and included the following: (i) Proteins that were purified with a telomere probe in an ALT- and a telomerase-positive cell line [14], (ii) proteins from the analysis of telomeric chromatin of telomerase-positive cells [38], (iii) proteins in close proximity to shelterin components [16, 17], (iv) proteins that affected ALT-associated PML nuclear bodies [23, 39], (v) deregulated proteins linked to telomere shortening [24], (vi) genes identified from telomerase activity signatures derived from gene expression data [40], (vii) telomerase regulators identified in a kinase screen and transcription factors compiled in a review [18, 19] and, (viii) a gene set with potential relevance to telomeres and the ALT pathway [41]. In addition, more than 1100 budding yeast genes were included in *TelNet*. For yeast, the initial gene list was obtained from the following sources: (i) Deletion screens identifying TL associated genes [26–28], (ii) post-senescent survivor screening after telomerase knockout [42], (iii) transcription factors of telomerase [43], and (iv) all human and budding yeast genes with a GO annotation containing the term “telo” [44].

**Table 1:** Screening studies and database information included in *TelNet* for identification of TM genes

Method	Organism / cell line	# genes/proteins	Ref.
Proteomics of isolated chromatin segments (PICh)	human / Wi38-VA13 HeLa 1.2.11	296	[14]
Quantitative telomeric chromatin isolation protocol (QTIP)	human / HeLa	34	[38]
Protein network analysis surrounding telomere repeat binding factors, TRF1, TRF2, and POT1 using dual-tag affinity purification in combination with multidimensional protein identification technology liquid chromatography - tandem mass spectrometry (MudPIT LC-MS/MS)	human / 293 T	211	[16]
Protein complementation assay (PCA/bimolecular fluorescent complementation (BiFC)) of shelterin compounds and ~ 12.000 candidate genes; GST-pulldown of FLAG-tagged genes	human / HTC75	339	[17]
siRNA mediated knockdown; APB formation	human / U2OS	29	[23, 39]
Proteomic analysis of deregulated genes upon telomere shortening caused by the telomerase inhibitor GRN163	human / SK-N-MC	99	[24]
QTRAP: kinase library screen	human / HeLa	109	[18]
Telomerase regulators affecting its transcription	human / various	53	[19]
Gene set with potential relevance to telomeres and the ALT pathway	human / various	297	[41]
Telomerase activity signature	human	43	[40]
GO annotation containing the term “telo”	human	245	[44]
Haploid deletion screen, telomere length	yeast	166	[26]
Telomere length-variation screen in deletion strains	yeast	138	[27]
Screen of DAmP collection	yeast	77	[28]
Telomerase null screen of yeast mutants	yeast	270	[42]
Telomerase regulators from the Yeastract database	yeast	35	[43]
GO annotation containing the term “telo”	yeast	192	[44]

To classify the relevance of a gene or corresponding protein for TM we introduced the three categories “predicted”, “screened” and “validated”. The factors collected from the above-mentioned screening or review sources were initially classified as “screened”. Genes with a suggested role in telomere maintenance but lacking experimentally validation were assigned with the TM significance “predicted”. Those with gene specific experimental evidence for a connection to telomere maintenance were ranked as “validated”. Orthologues of gene’s classified as “screened” or “validated” in one organism were included in the *TelNet* database as “predicted” in the other organism if no further information was available. In this manner, we compiled an initial list of human and budding yeast genes that was further curated and annotated manually.

### General information from external databases

For a standardized nomenclature [45], the converter system from DAVID Bioinformatics Resources (https://david.ncifcrf.gov/) [46] or the BioMarts tool from Ensembl [47] were used to provide gene and protein identifiers for Entrez, Hugo, Ensembl, Refseq and UniProt. To account for organism specific differences such as the lack of splicing isoforms in yeast or the absence of locus tags in human, the identifiers were selected differentially for each species. General gene information was retrieved from designated external databases and repositories, such as the National Center for Biotechnology Information (NCBI, https://www.ncbi.nlm.nih.gov) [48], HUGO Gene Nomenclature Committee (HGNC, http://www.genenames.org) [49], Ensembl (http://www.ensembl.org/index.html) [50, 51], and the Saccharomyces Genome Database (SGD, http://www.yeastgenome.org) [52]. The approved gene symbol, full name, and synonyms were taken from NCBI. UniProt [53] and Yeastmine [54] were consulted for the description of the cellular function in human and yeast, respectively, and assignment of orthologues was done with YeastMine. Based on the Gene Ontology (GO, http://www.geneontology.org) annotations [44] and in line with general biocuration guidelines [55] as well as SGD practice [56] we generated a list of cellular functions. Every gene was manually annotated with the respective term that was most representative for its cellular function. In this manner, general information for every gene entry was compiled from a variety of external databases.

### Telomere maintenance annotation with literature information and scoring

**Fig. 2:**
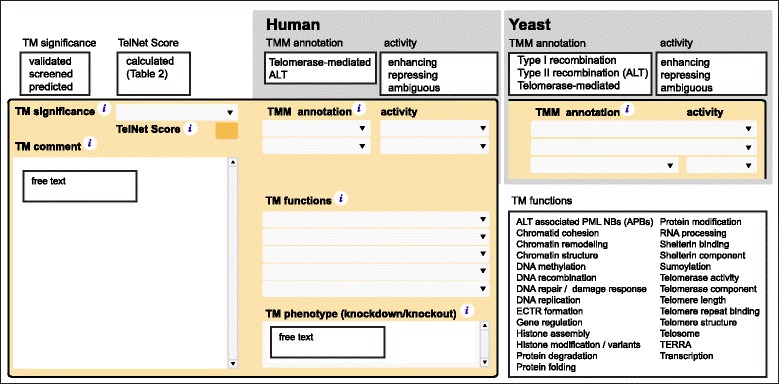
TM part of the *TelNet* gene card view. Annotation fields and possible entries for TM significance, associated *TelNet* score, TM comment, TMM annotation, TM functions and TM phenotype are depicted

Genes were further annotated with TM information from peer-reviewed literature for assigning them to functional categories (Fig. 2). Up to five TM functions of an assembled list that comprises molecular functions as well as cellular processes and structures with regard to TM can be selected. A knock-out or knock-down phenotype related to TM features such as alterations in TL, increased or decreased ALT hallmarks, or effects on telomerase was described as free-text in the field “TM phenotype”. Details from the literature were summarized in the field “TM comment”. To quantify the significance of a given gene for TM we introduced the *TelNet* score ranging from 1 (low) to 10 (high) that was automatically calculated from information entered into the *TelNet* database (Table 2). Scoring criteria included the cellular function, number and relevance of assigned TM functions and the amount of experimental data associated with the TM function of a given gene. Information on the protein's activity was collected in the “TMM annotation” field. For human genes, it was distinguished between “alternative lengthening of telomeres (ALT)” versus “telomerase-mediated” regulation with the associated activities “repressing”, “enhancing” or “ambiguous”. The latter refers to cases where literature information was inconsistent or was used for genes that were mentioned in the context of ALT or telomerase without further details of regulation activity. Budding yeast genes were annotated as survivors using “type I recombination” or “type II recombination” or associated with “telomerase-mediated” regulation. Thus, the annotation of a given factor in *TelNet* assigns it to ALT or a telomerase-mediated TMM and provides information on how it affects this process. Furthermore, the corresponding *TelNet* score provides an assessment of the significance of this assignment.

**Table 2:** Calculation of the *TelNet* score

Feature	Score
Identification in a TM related screen (added up if present in multiple screens)	1
TM significance is “validated”	3
If TM significance is “predicted”, the gene yields 10% of the orthologue’s *TelNet* score	0-1
Cellular function contains the term “telomere”	1.5
A TM function is assigned (added up for multiple functions)	0.5
TM function is “shelterin”, “telosome” or “telomerase”	0.5-2

The different features and their associated *TelNet* score are listed. The final value is calculated as the sum of the different entries and can reach a maximum value of 10

## Utility

### *TelNet* user interface

**Fig. 3:**
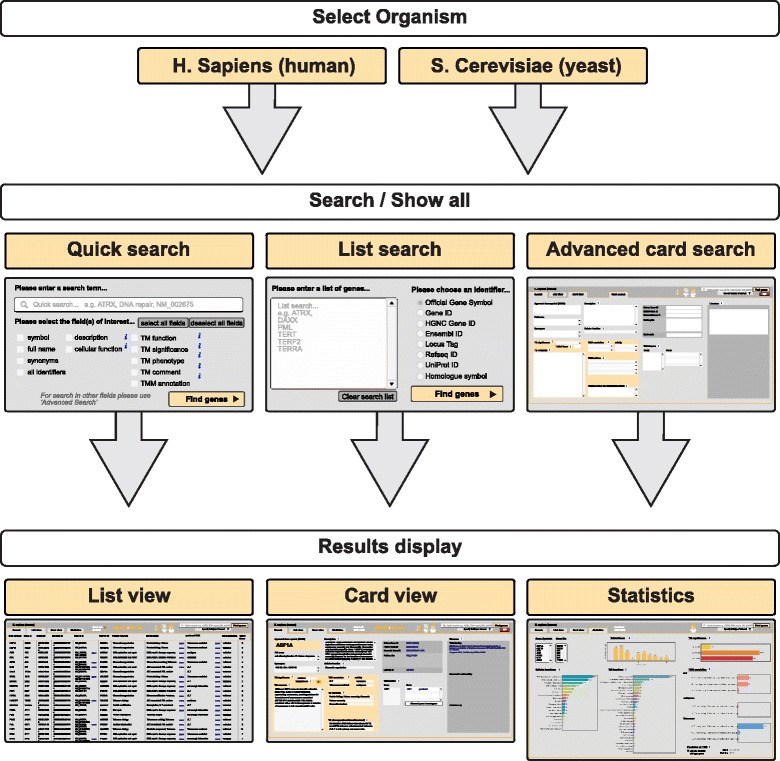
Typical *TelNet* workflow. *Top:* On the front page, the organism is selected. *Middle:* Three different search options, namely “quick search”, “list search” and “advanced card search” are available to retrieve a set of genes. *Bottom:* The resulting genes can be displayed as a scrollable list or as a series of single gene cards. In addition, an overview of the associated TM annotations is provided on the statistics page

On the start layout of *TelNet,* the user selects the organism, i.e. either *H. sapiens* or *S. cerevisiae* (Fig. 3). The default selection is *H. sapiens*. All genes can be browsed by clicking on the “show all” button. Furthermore, various search modes are available are described in more detail below. A navigation panel at the top allows switching between different views and returning to the front search page. Gene sets can be displayed as a scrollable list and the complete information of an individual gene is given by selecting the “card view”. A short explanation of each annotation field is given by clicking on the corresponding info button. Orthologous genes are connected via database hyperlinks. Furthermore, every gene is linked to selected publications.

### Search and statistics

The *TelNet* database can be used with three different search modes (Fig. 3) named “quick search”, “list search” and “advanced card search”. For a quick search throughout selected fields, one keyword can be entered into the search bar. If a user wishes to constrain the the results (e.g., to a gene symbol), the selection of fields can be adapted. By performing an advanced card search, the user can enter more and different search terms in respective fields. Furthermore, a complete list of gene identifiers can also be pasted into the list search. The organism and identifier provided are mandatory to perform a list search. Genes found are then displayed and can be selected for further analysis and TM network identification within *TelNet* or exported in various file formats. The statistics page gives a graphical overview over the distributions of various *TelNet* annotations such as a histogram of the *TelNet* score and the distribution of TM significance categories. Furthermore, *TelNet* statistics can be employed for a more detailed pathway analysis regarding TM functions. A predicted wild-type TMM is computed by evaluation of the TMM annotations retrieved. The wild-type phenotype of a given gene is used for predicting the likely active TMM for a set of genes. Every protein contributes with its *TelNet* score to one of the groups “ALT”, “telomerase-mediated” or “ambiguous”, which refers to its wild-type form. For example, a gene that is recurrently mutated in ALT positive tumors like *ATRX* (alpha thalassemia/mental retardation syndrome X-linked protein) would represent an ALT suppressor. It is thus classified as “telomerase-mediated” for the predicted TMM associated with its wild-type phenotype. The attribute “ambiguous” is used for genes lacking TMM information as well as genes with conflicting associations. Thus, *TelNet* informs about known and predicted TM features for the genes of interest via its different search and summary analysis tools.

### Application of *TelNet* for telomere maintenance analysis

The added value of *TelNet* in comparison to existing databases lies in the straightforward annotation of genes with respect to a TM function without pre-existing knowledge on the user side. For example, the Yeastract database lists 22 transcription factors (TFs) as “documented” regulators of the yeast *Est2* gene, encoding the telomerase catalytic subunit [43]. When submitting these TFs to the Saccharomyces Genome Database (SGD) with YeastMine all 22 genes were identified as transcription factors by the GO pathway analysis [54]. However, no enriched GO terms or publications related to telomeres/telomerase were returned because these TFs were not annotated with a telomere-associated GO term. In contrast, all 22 TFs were included in the *TelNet* database as *Est2* regulators.

**Fig. 4:**
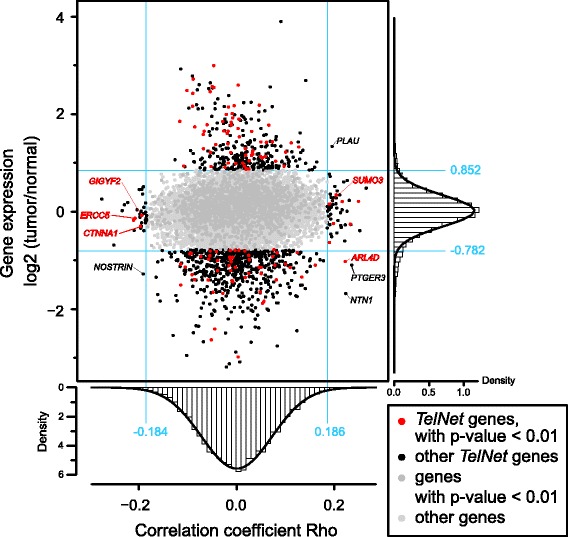
Application of *TelNet* for a correlation analysis of telomere length and gene expression. Scatter plot showing the log2 ratio (tumor/normal) of gene expression versus the Spearman correlation coefficient Rho for gene expression and telomere length. For histograms of Rho and log2 ratio a Gaussian fit is shown with significance values defined from the 1%-tail of the fit. Genes that were either significantly (*p* < 0.01) up- (log2 ratio > 0.852) or downregulated (log2 ratio < − 0.782) or significantly (*p* < 0.01) correlated (Rho > 0.186) or anti-correlated (Rho < − 0.184) were colored in black. Genes above the significance thresholds that were present in the *TelNet* database are shown in red color

The information provided by *TelNet* is particularly useful for the evaluation of gene lists obtained from large scale data sets as illustrated in the following for a pan-cancer correlation analysis of gene expression data with TL estimates. It is based on the cancer genome atlas (TCGA) study of Barthel et al. [40] and uses TL data calculated from whole genome sequencing (WGS) and gene expression data (stdata_2016_01_28, file uncv2.mRNAseq_RSEM_normalized_log2) downloaded via the firehose data repository (https://gdac.broadinstitute.org/). A reduced patient data set (*n* = 281) was selected that comprised all samples where non-malignant control samples of matching tumor tissue were available. In order to normalize for tissue- and age-specific effects, we calculated the ratio of tumor over normal tissue for TL and the corresponding log2 ratio for gene expression. For the two ratios, the spearman correlation coefficient was computed. For 87 genes, a significant correlation (*p* < 0.01 and − 0.184 < Rho > 0.186) of TL with gene expression was found and 940 genes were differentially expressed (*p* < 0.01 and < − 0.782 log2 ratio > 0.852) (Fig. 4, Additional file 1: Table S1). For 5 genes both a correlation of TL and gene expression was found, namely *NTN1*, *PTGER3*, *ARL4D*, *PLAU* and *NOSTRIN*. It is noted that most of the tumor samples had shorter telomeres than the respective normal control sample. This could be the result of a higher tumor proliferation rate being only partly compensated by the active TMM. This confounding factor as well as the tissue specific expression programs in the different tumor entities are likely to lead to false negative results. For example, *TERT* (telomerase reverse transcriptase) expression did not show a significant correlation with TL. Thus, it might be also informative to examine deregulated genes that did not display an (anti-)correlation with TL with respect to potential TM activities.

To further analyze the 1022 genes for which correlations or deregulations on the gene expression level were detected, we consulted the HumanMine database and its GO enrichment analysis [57]. The enriched GO terms did not return a telomere-related pathway. Without an enrichment threshold, 13 genes (*RAD51*, *CCNE1*, *BRCA2*, *HIST1H4H*, *RECQL4*, *RFC4*, *FEN1*, *EXO1*, *BLM* and *HIST2H4A, PPARG*, *KLF4* and *PARM1*) were annotated with one of the GO terms “telomere maintenance”, “telomere organization” or “regulation of telomerase activity”. In contrast, we retrieved a set of 132 genes when using the *TelNet* “list search” option (Additional file 1: Table S1). *TelNet* finds more genes, because it includes homology assignments in both directions (30 from the 132 genes have a yeast homolog with a TM phenotype) as well as genes that do not have a GO term related to telomeres but have telomere related activities according to the papers referenced in *TelNet*. Out of the 132 genes found in the *TelNet* database, 12 showed a significant (anti-)correlation (0.186 > Rho < − 0.184) between TL and gene expression (Table 3): Only one gene, *ARL4D* (ADP-ribosylation factor-like protein 4D), additionally had a significant deregulation of gene expression in tumors (log2 ratio = − 1.02). *ARL4D* was included in *TelNet* since the deletion mutant of its yeast orthologue *ARF1* (ADP ribosylation factor 1) has shorter telomeres than the wild-type reference [27, 42]. Furthermore, 4 genes of those listed in Table 3 were annotated in *TelNet* as “screened” or “validated” and had *TelNet* scores > 1 (Table 3). *CTNNA1* (catenin alpha 1) and *GIGYF2* (GRB10 interacting GYF protein 2) were found in a screen for genes that were upregulated upon telomere shortening [24]. This finding is consistent with the phenotype of the budding yeast homologue of *GIGYF2* (*SYH1*), the deletion of which has been shown to lead to a TL increase [26]. In addition, *SUMO3* (small ubiquitin-like modifier 3) and *ERCC5* (excision repair 5 endonuclease) were included in *TelNet* as having a validated human TM phenotype. The SUMO3 domain is attached to key proteins of the ALT pathway and shows a positive correlation with TL. Sumoylation of PML and shelterin compounds are known to be essential for the formation of PML nuclear bodies and APBs [23, 58]. The ERCC5 endonuclease is involved in DNA recombination and repair by annealing single-stranded DNA. Furthermore, ERCC5 regulates the activity of the Werner syndrome helicase (WRN) [59] that is required for telomere maintenance in some ALT cell lines [60] and is involved in telomeric D-loop digestion in ALT cells [61]. We conclude from this *TelNet* supported analysis that a further investigation of ARL4D, GIGYF2, CTNNA1, SUMO3 and ERCC5 with respect to their role for telomere maintenance in tumor cells might be warranted.

**Table 3:** Genes with (anti-)correlations between telomere length and gene expression listed in *TelNet*

Gene	Rho	*p*-value (Rho)	log2 expr. Ratio	*p*-value (expr.)	TM information from *TelNet*	*TelNet score*
*PRPS2*	0.25	4.7E-04	0.21	4.4E-01	Predicted from budding yeast homologue	1
*GATA3*	0.23	1.1E-03	−0.26	3.6E-01	Predicted from budding yeast homologue	1
*ARL4D*	0.22	1.8E-03	−1.02	8.6E-04	Predicted from budding yeast homologue	1
*PDK3*	0.22	2.4E-03	0.21	4.5E-01	Predicted from budding yeast homologue	1
*FAM58A*	0.20	4.2E-03	0.35	2.3E-01	Predicted from budding yeast homologue	1
*MRPL34*	0.20	5.4E-03	−0.25	3.8E-01	Predicted from budding yeast homologue	1
*TSPYL5*	0.19	7.7E-03	−0.63	3.5E-02	Predicted from budding yeast homologue	1
*SUMO3*	0.19	7.8E-03	0.16	5.4E-01	Validated in human	6
*GIGYF2*	−0.19	6.6E-03	−0.08	7.2E-01	Human screen for telomere shortening	2
*PPM1D*	−0.20	5.9E-03	−0.30	2.9E-01	Predicted from budding yeast homologue	1
*CTNNA1*	−0.21	3.6E-03	−0.14	5.9E-01	Human screen for telomere shortening	2
*ERCC5*	−0.21	3.1E-03	−0.18	5.0E-01	Validated in human	5

In a pan-cancer analysis, genes were identified that showed a significant (anti-)correlation (correlation coefficient Rho < − 0.184 or Rho > 0.186 and, *p* < 0.01) between telomere length and gene expression. For the calculations, the values for the ratios of tumor over normal tissue were used. The table shows those genes that were listed in the *TelNet* database with their TM information and *TelNet* scores

## Discussion

The *TelNet* database offers a fast identification of genes from different “omics” approaches, e.g., WGS and RNA-seq data with respect to their potential activities for telomere maintenance. It is designed as an open-ended database for the collection of TM relevant genes in different organisms. An extension of *TelNet* in its next release will include compilations from TM genes from two additional organisms, namely *S. pombe* and *M. musculus*. Accordingly, new information on telomere maintenance will be added continuously. We encourage other researchers working on telomeres to communicate suggestions for missing genes or additional information on already existing entries via the link integrated in the database to telnet@dkfz.de.

A gene set derived from a preceding bioinformatics analysis pipeline can be directly used for a *TelNet* list search to get more detailed insight on the corresponding TM associated genes. Possible TM links can be explored in an iterative manner. This approach is particularly useful for the large data sets generated in current genome and transcriptome sequencing studies as illustrated here for the TCGA pan-cancer data analysis from ref. [40]. In a similar manner, a current study of the ICGC (international cancer genome consortium) made use of *TelNet* to characterize genomic features of the active TM in cancer [62]. It is noted that some well-established associations like mutations in *ATRX* and *DAXX* (death-domain associated protein) for ALT as well as *TERT* promoter mutations for telomerase-positive cells are absent in many tumor samples. Thus, one would expect that for these cases the mutation status of a given cancer sample and its active TM are linked via other genes, possibly as a combination of multiple factors. Consistent with this expectation, an integrative genome and transcriptome analysis of leiomyosarcoma applied *TelNet* for the TMM annotation and identified recurrent mutations in *RBL2* (RB transcriptional corepressor like 2) and *SP100* (SP100 nuclear antigen) as linked to ALT [63].

## Conclusion

The gene annotations provided by *TelNet* largely facilitate a distinction between different TM mechanisms for a gene set of interest by providing corresponding functional terms and a significance ranking. With these features, *TelNet* supports the identification of TM networks in various ways. As illustrated here by an exemplary application, *TelNet* can be integrated into the annotation of genes identified from bioinformatics analysis pipelines to determine possible connections with TM networks. Accordingly, we anticipate that *TelNet* will prove to be a helpful analysis tool for revealing this type of correlations and will support the identification of active TM networks in different tumor entities.

## Additional file

Additional file 1: The table lists the 1022 genes identified in the pan-cancer analyis described in the context of Fig. 4. These genes have either significant changes of gene expression in tumor over normal cells (*p*-value < 0.01, log2 ratio below − 0.782 or above 0.852) or an (anti-) correlation of gene expression and telomere length ratios (*p*-value < 0.01, Rho below − 0. 295 or above 0.186). In addition, TM information is given for those genes that were present in the *TelNet* database. (XLSX 152 kb)

## Abbreviations

ALT: Alternative lengthening of telomeres
APB: ALT-associated PML nuclear bodies
ARL4D: ADP ribosylation factor-like GTPase 4D
ATRX: Alpha thalassemia/mental retardation syndrome X-linked protein
CTNNA1: Catenin alpha 1
ERCC5: Excision repair 5 endonuclease
GIGYF2: GRB10 interacting GYF protein 2
GO: Gene ontology
PML: Promyelocytic leukemia
SUMO3: Small ubiquitin-like modifier 3
TCGA: The cancer genome atlas
TERT: Telomerase reverse transcriptase
TF: Transcription factor
TL: Telomere length
TM: Telomere maintenance
TMM: Telomere maintenance mechanism
WGS: Whole genome sequencing

## References

[1] de Lange T (2005). Shelterin: the protein complex that shapes and safeguards human telomeres. Genes Dev.

[2] Blackburn EH, Epel ES, Lin J (2015). Human telomere biology: a contributory and interactive factor in aging, disease risks, and protection. Science.

[3] Denchi EL, de Lange T (2007). Protection of telomeres through independent control of ATM and ATR by TRF2 and POT1. Nature.

[4] Palm W, de Lange T (2008). How shelterin protects mammalian telomeres. Annu Rev Genet.

[5] Lazzerini-Denchi E, Sfeir A (2016). Stop pulling my strings - what telomeres taught us about the DNA damage response. Nat Rev Mol Cell Biol.

[6] Shay JW (2016). Role of telomeres and telomerase in aging and Cancer. Cancer Discov.

[7] Heidenreich B, Rachakonda PS, Hemminki K, Kumar R (2014). TERT promoter mutations in cancer development. Curr Opin Genet Dev.

[8] Peifer M, Hertwig F, Roels F, Dreidax D, Gartlgruber M, Menon R, Kramer A, Roncaioli JL, Sand F, Heuckmann JM (2015). Telomerase activation by genomic rearrangements in high-risk neuroblastoma. Nature.

[9] Valentijn LJ, Koster J, Zwijnenburg DA, Hasselt NE, van Sluis P, Volckmann R, van Noesel MM, George RE, Tytgat GA, Molenaar JJ (2015). TERT rearrangements are frequent in neuroblastoma and identify aggressive tumors. Nat Genet.

[10] Cesare AJ, Reddel RR (2010). Alternative lengthening of telomeres: models, mechanisms and implications. Nat Rev Genet.

[11] Heaphy CM, de Wilde RF, Jiao Y, Klein AP, Edil BH, Shi C, Bettegowda C, Rodriguez FJ, Eberhart CG, Hebbar S (2011). Altered telomeres in tumors with ATRX and DAXX mutations. Science.

[12] Dilley RL, Greenberg RA (2015). ALTernative telomere maintenance and Cancer. Trends Cancer.

[13] Sobinoff AP, Pickett HA (2017). Alternative lengthening of telomeres: DNA repair pathways converge. Trends Genet.

[14] Dejardin J, Kingston RE (2009). Purification of proteins associated with specific genomic loci. Cell.

[15] Grolimund L, Aeby E, Hamelin R, Armand F, Chiappe D, Moniatte M, Lingner J (2013). A quantitative telomeric chromatin isolation protocol identifies different telomeric states. Nat Commun.

[16] Giannone RJ, McDonald HW, Hurst GB, Shen RF, Wang Y, Liu Y (2010). The protein network surrounding the human telomere repeat binding factors TRF1, TRF2, and POT1. PLoS One.

[17] Lee OH, Kim H, He Q, Baek HJ, Yang D, Chen LY, Liang J, Chae HK, Safari A, Liu D (2011). Genome-wide YFP fluorescence complementation screen identifies new regulators for telomere signaling in human cells. Mol Cell Proteomics.

[18] Cerone MA, Burgess DJ, Naceur-Lombardelli C, Lord CJ, Ashworth A (2011). High-throughput RNAi screening reveals novel regulators of telomerase. Cancer Res.

[19] Ramlee MK, Wang J, Toh WX, Li S (2016). Transcription regulation of the human telomerase reverse transcriptase (hTERT) gene. Genes (Basel).

[20] Yeager TR, Neumann AA, Englezou A, Huschtscha LI, Noble JR, Reddel RR (1999). Telomerase-negative immortalized human cells contain a novel type of promyelocytic leukemia (PML) body. Cancer Res.

[21] Nabetani A, Ishikawa F (2011). Alternative lengthening of telomeres pathway: recombination-mediated telomere maintenance mechanism in human cells. J Biochem.

[22] Chung I, Osterwald S, Deeg KI, Rippe K (2012). PML body meets telomere: the beginning of an ALTernate ending?. Nucleus.

[23] Osterwald S, Deeg KI, Chung I, Parisotto D, Wörz S, Rohr K, Erfle H, Rippe K (2015). PML induces compaction, TRF2 depletion and DNA damage signaling at telomeres and promotes their alternative lengthening. J Cell Sci.

[24] Uziel O, Yosef N, Sharan R, Ruppin E, Kupiec M, Kushnir M, Beery E, Cohen-Diker T, Nordenberg J, Lahav M (2015). The effects of telomere shortening on cancer cells: a network model of proteomic and microRNA analysis. Genomics.

[25] Kupiec M (2014). Biology of telomeres: lessons from budding yeast. FEMS Microbiol Rev.

[26] Askree SH, Yehuda T, Smolikov S, Gurevich R, Hawk J, Coker C, Krauskopf A, Kupiec M, McEachern MJ (2004). A genome-wide screen for Saccharomyces cerevisiae deletion mutants that affect telomere length. Proc Natl Acad Sci U S A.

[27] Gatbonton T, Imbesi M, Nelson M, Akey JM, Ruderfer DM, Kruglyak L, Simon JA, Bedalov A (2006). Telomere length as a quantitative trait: genome-wide survey and genetic mapping of telomere length-control genes in yeast. PLoS Genet.

[28] Ungar L, Yosef N, Sela Y, Sharan R, Ruppin E, Kupiec M (2009). A genome-wide screen for essential yeast genes that affect telomere length maintenance. Nucleic Acids Res.

[29] Lippuner AD, Julou T, Barral Y (2014). Budding yeast as a model organism to study the effects of age. FEMS Microbiol Rev.

[30] Lundblad V (2002). Telomere maintenance without telomerase. Oncogene.

[31] Lundblad V, Blackburn EH (1993). An alternative pathway for yeast telomere maintenance rescues est1- senescence. Cell.

[32] Chen Q, Ijpma A, Greider CW (2001). Two survivor pathways that allow growth in the absence of telomerase are generated by distinct telomere recombination events. Mol Cell Biol.

[33] Teng SC, Zakian VA (1999). Telomere-telomere recombination is an efficient bypass pathway for telomere maintenance in Saccharomyces cerevisiae. Mol Cell Biol.

[34] Podlevsky JD, Bley CJ, Omana RV, Qi X, Chen JJ (2008). The telomerase database. Nucleic Acids Res.

[35] Huang Z, Ma L, Wang Y, Pan Z, Ren J, Liu Z, Xue Y (2015). MiCroKiTS 4.0: a database of midbody, centrosome, kinetochore, telomere and spindle. Nucleic Acids Res.

[36] Luo Z, Dai Z, Xie X, Feng X, Liu D, Songyang Z, Xiong Y. TeloPIN: a database of telomeric proteins interaction network in mammalian cells. Database (Oxford). 2015;2015(0):bav018–8. 10.1093/database/bav018 PMC4365144 25792605

[37] Gowthaman R, Krishnamoorthy S, Nandakumar RD, Ayyarappan V (2007). TeCK database: a comprehensive collection of telomeric and centromeric sequences with their associated proteins. Bioinformation.

[38] Grolimund L, Aeby E, Hamelin R, Armand F, Chiappe D, Moniatte M, Lingner J (2013). A quantitative telomeric chromatin isolation protocol identifies different telomeric states. Nat Commun.

[39] Jiang WQ, Zhong ZH, Henson JD, Reddel RR (2007). Identification of candidate alternative lengthening of telomeres genes by methionine restriction and RNA interference. Oncogene.

[40] Barthel FP, Wei W, Tang M, Martinez-Ledesma E, Hu X, Amin SB, Akdemir KC, Seth S, Song X, Wang Q (2017). Systematic analysis of telomere length and somatic alterations in 31 cancer types. Nat Genet.

[41] Lovejoy CA, Li W, Reisenweber S, Thongthip S, Bruno J, de Lange T, De S, Petrini JH, Sung PA, Jasin M (2012). Loss of ATRX, genome instability, and an altered DNA damage response are hallmarks of the alternative lengthening of telomeres pathway. PLoS Genet.

[42] Hu Y, Tang HB, Liu NN, Tong XJ, Dang W, Duan YM, Fu XH, Zhang Y, Peng J, Meng FL (2013). Telomerase-null survivor screening identifies novel telomere recombination regulators. PLoS Genet.

[43] Teixeira MC, Monteiro PT, Guerreiro JF, Goncalves JP, Mira NP, dos Santos SC, Cabrito TR, Palma M, Costa C, Francisco AP (2014). The YEASTRACT database: an upgraded information system for the analysis of gene and genomic transcription regulation in Saccharomyces cerevisiae. Nucleic Acids Res.

[44] Gene Ontology C (2015). Gene ontology consortium: going forward. Nucleic Acids Res.

[45] Klionsky DJ, Bruford EA, Cherry JM, Hodgkin J, Laulederkind SJ, Singer AG (2012). In the beginning there was babble. Autophagy.

[46] da Huang W, Sherman BT, Lempicki RA (2009). Systematic and integrative analysis of large gene lists using DAVID bioinformatics resources. Nat Protoc.

[47] Kinsella RJ, Kahari A, Haider S, Zamora J, Proctor G, Spudich G, Almeida-King J, Staines D, Derwent P, Kerhornou A (2011). Ensembl BioMarts: a hub for data retrieval across taxonomic space. Database (Oxford).

[48] Brown GR, Hem V, Katz KS, Ovetsky M, Wallin C, Ermolaeva O, Tolstoy I, Tatusova T, Pruitt KD, Maglott DR (2015). Gene: a gene-centered information resource at NCBI. Nucleic Acids Res.

[49] Yates B, Braschi B, Gray KA, Seal RL, Tweedie S, Bruford EA (2017). Genenames.Org: the HGNC and VGNC resources in 2017. Nucleic Acids Res.

[50] Aken BL, Achuthan P, Akanni W, Amode MR, Bernsdorff F, Bhai J, Billis K, Carvalho-Silva D, Cummins C, Clapham P (2017). Ensembl 2017. Nucleic Acids Res.

[51] Aken BL, Ayling S, Barrell D, Clarke L, Curwen V, Fairley S, Fernandez Banet J, Billis K, Garcia Giron C, Hourlier T, et al. The Ensembl gene annotation system. Database (Oxford). 2016;2016:baw093. 10.1093/database/baw093 PMC4919035 27337980

[52] Cherry JM (2015). The Saccharomyces genome database: a tool for discovery. Cold Spring Harb Protoc.

[53] The UniProt C (2017). UniProt: the universal protein knowledgebase. Nucleic Acids Res.

[54] Balakrishnan R, Park J, Karra K, Hitz BC, Binkley G, Hong EL, Sullivan J, Micklem G, Cherry JM (2012). YeastMine--an integrated data warehouse for Saccharomyces cerevisiae data as a multipurpose tool-kit. Database (Oxford).

[55] Poux S, Gaudet P (2017). Best practices in manual annotation with the gene ontology. Methods Mol Biol.

[56] Hong EL, Balakrishnan R, Dong Q, Christie KR, Park J, Binkley G, Costanzo MC, Dwight SS, Engel SR, Fisk DG (2008). Gene ontology annotations at SGD: new data sources and annotation methods. Nucleic Acids Res.

[57] Smith RN, Aleksic J, Butano D, Carr A, Contrino S, Hu F, Lyne M, Lyne R, Kalderimis A, Rutherford K (2012). InterMine: a flexible data warehouse system for the integration and analysis of heterogeneous biological data. Bioinformatics.

[58] Chung I, Leonhardt H, Rippe K (2011). De novo assembly of a PML nuclear subcompartment occurs through multiple pathways and induces telomere elongation. J Cell Sci.

[59] Trego KS, Chernikova SB, Davalos AR, Perry JJ, Finger LD, Ng C, Tsai MS, Yannone SM, Tainer JA, Campisi J (2011). The DNA repair endonuclease XPG interacts directly and functionally with the WRN helicase defective in Werner syndrome. Cell Cycle.

[60] Gocha AR, Acharya S, Groden J (2014). WRN loss induces switching of telomerase-independent mechanisms of telomere elongation. PLoS One.

[61] Opresko PL, Otterlei M, Graakjaer J, Bruheim P, Dawut L, Kolvraa S, May A, Seidman MM, Bohr VA (2004). The Werner syndrome helicase and exonuclease cooperate to resolve telomeric D loops in a manner regulated by TRF1 and TRF2. Mol Cell.

[62] Sieverling L, Hong C, Koser SD, Ginsbach P, Kleinheinz K, Hutter B, Braun DM, Cortes-Ciriano I, Xi R, Kabbe R, et al. Genomic footprints of activated telomere maintenance mechanisms in cancer. Nat Commun. 2018:in press, preprint; 10.1101/157560. 10.1038/s41467-019-13824-9 PMC7002710 32024817

[63] Chudasama P, Mughal SS, Sanders MA, Hubschmann D, Chung I, Deeg KI, Wong SH, Rabe S, Hlevnjak M, Zapatka M (2018). Integrative genomic and transcriptomic analysis of leiomyosarcoma. Nat Commun.

